# Enhanced Analysis of Carcinogens and Nutritional Profile of *Vitis vinifera* by Employing Pulsed Electric Field

**DOI:** 10.1002/fsn3.4590

**Published:** 2024-11-18

**Authors:** Zahra Batool, Dur e Sameen, Ding Wen, Shanshan Hu, Ume Roobab, Bing Li, Rana Muhammad Adil, Bairong Shen

**Affiliations:** ^1^ Center of High Altitude Medicine West China Hospital, Sichuan University Chengdu China; ^2^ College of Food Science Sichuan Agricultural University Yaan China; ^3^ Department of Clinical Research Management, Department of Laboratory Medicine West China Hospital, Sichuan University Chengdu China; ^4^ School of Food Science and Technology South China University of Technology Guangzhou China; ^5^ National Institute of Food Science and Technology University of Agriculture Faisalabad Pakistan

**Keywords:** food processing, Maillard reaction, nutrition, pulsed electric field, SPME‐GC‐TQ/MS

## Abstract

Solid Phase Microextraction‐Gas Chromatography Triple Quadrupole Mass Spectrometry (SPME‐GC‐TQ/MS) was optimized and validated to specifically analyze aldehydes and furans after drying *Vitis vinifera grape variety* by conventional as well as modern pre‐drying technique i.e. pulsed electric field (PEF). Analytical method was validated in terms of linearity (*R* ^2^), recovery (%), relative standard deviation (% RSD), limit of detection (LOD) and limit of quantification (LOQ). Sample pre‐treatment with PEF (1 kV/cm) followed by drying at 65°C produced dried product with fewer chemical changes in the composition than convective hot air drying. Meanwhile, nutritional parameters including polyphenolics, amino acids, fatty acids and sugars profiles were also extensively investigated to get deeper insights into the effect of drying treatments on the nutritional quality of dried product. Conclusively, sample pre‐treatment on relatively lower PEF voltage, followed by drying at minimum temperature with longer time duration can preserve nutritional quality of product by forming less harmful compounds.

## Graphical abstract

This research has found a wise combination of modern pre‐drying technique with conventional drying method to preserve the nutritional profile of dried product by minimum formation of undesired harmful compounds.

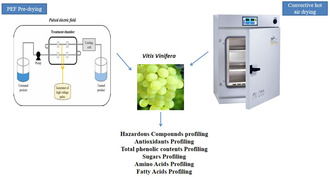

## 1 Introduction

*Vitis vinifera* L. is a popular grapevine variety that is cultivated worldwide (OIV 2017). According to the International Organization of Vine and Wine (OIV), *Vitis vinifera* L. is the only top white variety that experienced a significant increase (> 3%) in annual changes in vineyard area worldwide between 2000 and 2015 (OIV 2017). Carbohydrates are essential for determining the quality of raisins, which are formed in *Vitis vinifera* using various drying techniques and changes their conformation to their different derivatives (Wang et al. 2017). Meanwhile, acid hydrolysis, Maillard reaction, and caramelization are the most important chemical reactions that affect carbohydrates during drying. Volatile compounds in raisins, such as furans and pyrazine compounds, can come from fresh grapes or be produced during drying. Maillard reaction can produce roasted aromas (Toci and Farah 2014), whereas acids and aldehydes can result from the auto‐oxidation of unsaturated lipids (Whitfield and Mottram 1992). Different chemical reactions can significantly affect the final product quality and its nutritional profile depending upon drying temperature, pH of the fruit, and applied drying process (Wang et al. 2017). Therefore, there is a need to acquire better understanding of these chemical reactions, as well as their final products must be acquired to optimize the drying conditions (Wang et al. 2017). Furthermore, it is thought that these compounds may come from the heat treatment of the fruit, enzymatic degradation in the first stage of heating, or chemical degradation of sugars and amino acids. Wang et al. (2017) showed that the sugar degradation reaction was associated with the presence of furfural, acetylfuran and methylfurfural, in raisins. However, little work has been done to monitor changes in the volatile ingredients of food during the application of different drying treatments, particularly modern drying treatment, under optimized conditions.

Pulsed electric fields (PEF) drying is a novel technological opportunity for food processing and preservation. The typical electric field intensity in a PEF treatment ranges from 1 to 80 kV cm^−1^ with a pulse duration of micro‐to milliseconds (Arshad et al. 2021). PEF technology has been introduced as a nonthermal treatment for inactivation of microorganisms (Roobab et al. 2018) and enzymes (Roobab et al. 2022), with the purpose of achieving better preservation of food color, texture, flavor, and nutritional value, with respect to the traditional thermal processing methods (Ranjha et al. 2021). While some studies have discussed the potential application of PEF technologies in wineries (Morata et al. 2017; Piergiorgio et al. 2018).

Efficient analysis of analytical compounds is mandatory in order to get accurate information about the applied processing conditions, such as hot air drying or PEF. Solid Phase Microexraction accompanied with Gas Chromatography Mass Spectrometry (SPME‐GCMS) is very efficient analytical method to determine prevalence of volatile compounds including furans and aldehydes in different samples (Wang et al. 2017; Batool et al. 2020; Condurso, Cincotta, and Verzera 2018). However, the efficiency of analysis depends on the optimization of different parameters of analytical instrument while developing the analytical method for accurate determination of formed analytes in the sample matrix. Meanwhile, the nutritional profile of sample including sugars, lipids, polyphenols, fatty acids, amino acids are also very important in order to get deep insights about changes in the sample after being processed.

The hypothesis was to firstly develop and optimize sensitive SPME GC‐TQ/MS method for analysis of particularly aldehydes and furan derivatives formed during drying process of *Vitis vinifera* by optimizing best sample extraction, separation and analysis conditions. The optimized method was validated in terms of all validation parameters including linearity (*R* ^2^), recovery (%), repeatability in terms of relative standard deviation (%) including intra‐day and inter‐day precision, limit of detection (LOD) and limit of quantification (LOQ). Then we further focused on drying methods on our sample, and employed conventional hot air drying (solely temperature effect) and pre‐drying with modern drying technique, i.e. PEF followed by minimum temperature effect for drying. Different processed samples were then analyzed by optimized SPME‐GC‐TQ/MS method and their differently formed compounds were quantified in depth. Meanwhile, analysis of nutritional changes is also mandatory in order to get deeper insights about effect of physical changes of drying process on the sample matrix. Therefore, we further evaluated the changes in their polyphenolic profile, including antioxidant activity, phenolic content, then amino acid, sugar and fatty acids profile deeply for optimizing the best drying conditions in order to get minimum loss of nutrients in the dried product.

## 2 Materials and Chemicals

The following reagents were purchased from Sigma Aldrich (St. Louis, USA) including furan (99%), furfural (99%), 2‐pentylfuran (99%, 2‐PF), Hexanal (99%), Heptanal (98.9%), Pentanal (99%), Nonanal (99%), Furan 2‐ethyl‐ 5 methyl (99%), Furan 2‐Propyl (99%), 3‐Furaldehyde (98%), 5‐Hydroxymethylfurfural (≥ 98%), 1‐pentanone 2‐ furanyl (≥ 98%), salfosylicilic acid, high purity water, sodium chloride (99%), 50/30 um carboxen/divinylbenzene/polysimethylsiloxane (CAR/DVB/PDMS) fiber with manual holder (Supelco, Bellefonte, PA, USA), Folin–Ciocalteu reagent, 2,2‐diphenyl‐1‐picrylhydrazyl (DPPH), 95% methanol, 95% ethanol, acetonitrile, lead acetate, potassium hydroxide, n‐Hexane, 3‐dexyglucosone (75%), O‐phenylenediamine (98%), Diethylenetria minopentaacetic acid (98%), 2‐methylquinoxaline (97%), and formic acid. Disodium hydrogen phosphate anhydrous was obtained from Aladine Chemicals. Co (Shanghai, China), while purified water was obtained from Waters (St. Louis, USA). All sample bottles, syringe filters, and filter papers were purchased from Aladine Chemicals. Co (Shanghai, China). Standard stock solutions of 500 ng/g for each analyte were prepared in analytical‐grade water and stored at 4°C. Calibration standard curves were constructed for all compounds, and the quantification of all compounds was performed using their matrix matched calibration curves.

### 2.1 Convective Hot Air‐Drying Procedure

*Vitis vinifera* white Malaga grape varieties were selected for the drying experiment. A convective drying oven was then employed to dry washed samples at three different temperatures, 65°C, 70°C, and 75°C. These temperatures were chosen to identify the optimal drying conditions for accurate representation of the drying process. The samples were dried until the desired moisture content, ranging from 20% to 25% dry basis, was achieved. The drying experiments were conducted with a relative humidity and air velocity of approximately 5 m/s.

### 2.2 Pre‐Drying With Pulsed Electric Field

This experiment involved the application of pulsed electric field to *Vitis vinifera* grapes using the EX‐1900 machine, with voltage strengths of 1, 3, and 5 kV/cm. The Tektronix TBA1102B Digital Oscilloscope was utilized to generate the impulse. Following pre‐treatment with different voltages, separately, the grapes were further dried by hot air‐drying oven at 65°C. The electric field intensity was calculated by dividing the applied electric field by the sample displacement (kV/cm), and specific energy intake (kJ/kg) was determined using the following equation.

$$\text{Wspec}=U2^{*}C^{*}n/2m,$$

where *n* is the number of pulses, *m* is mass of treated sample (kg), *U* is voltage (kV), *C* is capacitance (1 μF).

The obtained intensity for each applied voltage per 2 g of sample for 5 given pulses are in the following

$$\text{Wspec}=12.5\;\mathrm{at}\;\left(5\;\mathrm{kV}/\mathrm{cm}\right)$$

$$\text{Wspec}=7.5\;\mathrm{at}\;\left(3\;\mathrm{kV}/\mathrm{cm}\right)$$

$$\text{Wspec}=2.5\;\mathrm{at}\;\left(1\;\mathrm{kV}/\mathrm{cm}\right)$$

### 2.3 Moisture Reduction Ratio Formation

The moisture content of the samples was determined by weighing them at hourly intervals, and a moisture reduction curve was generated for each drying treatment. Convective hot air drying, and PEF pre‐treatment were utilized to produce the resulting curves in Figures S1 and S2 respectively, illustrating the reduction in moisture content at various drying times and temperatures.

The equation for moisture reduction ratio formation

$$\mathrm{MR}=M_{t}–M_{e}/M_{0}–M_{e}$$

Where, M_*t*_ is the moisture content at drying time *t* (dry basis), M_*e*_ is the equilibrium moisture content, M_0_ is the initial moisture content.

### 2.4 Sample Preparation

The 5 g sample was mechanically blended and diluted with 5 mL water to ensure a homogeneous sample and prevent blending difficulties.

### 2.5 Head Space Solid Phase Microextraction (HS‐SPME)

A 50/30 μm carboxen/divinylbenzene/polydimethylsiloxane (CAR/DVB/PDMS) fiber with 8.5 cm length was used to extract analytes under various conditions, including extraction time (15, 25, and 35 min), temperature (40°C, 45°C, 50°C, and 55°C), NaCl concentration (10%, 15%, 20%, and 25% w/v), stirring speed (500, 600, 700, and 800 rpm), and maceration time (ranging from 1 to 6 h). The aim was to optimize these conditions for the best extraction efficiency. Through preliminary experiments, each parameter was tested under different conditions. The most effective conditions were selected for the main experiment. Among these, one extraction condition yielded the highest sensitivity for analyte detection across all parameters and was therefore used for the remainder of the study. Once these optimal conditions were identified, the experiment proceeded smoothly.

For sample preparation, 5 mL of supernatant from each test sample was added to a 20 mL headspace vial, along with 2.5 mL of a 15% NaCl solution. Water was then added to bring the total volume to 10 mL. The vials were equipped with a miniature valve that allowed the fiber to be introduced without puncturing a septum, preventing contamination from septum exudation. The heater temperature was set to 45°C, and the vials were placed on the heater one by one to allow analyte extraction. A 50/30 μm CAR/DVB/PDMS fiber (Supelco, Bellefonte, PA, USA) with 8.5 cm length, mounted on a manual holder, was used. Before use, the fiber was conditioned in a gas chromatography (GC) inlet at 250°C for 30 min, as per the manufacturer's instructions. Each sample was equilibrated on the heater for 25 min at 45°C before extraction. The analytes were extracted with the fiber for 25 min while continuously stirring at 600 rpm using a small magnetic stir bar inside the vial. After extraction, the fiber was removed from the vial and inserted into the splitless injector of a GC–MS, maintained at 250°C, for complete desorption of the volatiles over 5 min.

### 2.6 GC‐TQ/MS

An Agilent technologies 7890B Gas Chromatograph interfaced with a 7000C Triple Quadruple Mass Spectrometer (GC‐TQ/MS) equipped with GC capillary column, DB Wax column (30 m × 0.25 mm × 0.25 μm) or HP‐5MS column (30 m × 0.25 mm × 0.5 μm) separately, were compared for their efficiency for analytes separation one by one, firstly by combining GC‐TQ/MS with DB Wax columns and then GC‐TQ/MS with HP‐5MS column; however, HP‐5MS column has provided better separation results with appropriate retention times and most of compounds eluted on HP‐5MS column with relatively higher peaks which were even not shown on DB Wax column. Therefore, we decided to further use HP‐5MS column for all subsequent analysis of volatiles in this study.

With a HP‐5MS column (30 m × 0.25 mm × 0.25 μm) was utilized to separate and analyze the volatiles present in dried raisins. Helium was employed as the carrier gas at a constant flow rate of 1 mL/min, while a split GC inlet mode (5:1) was used to introduce the volatiles from an SPME needle into the GC at approximately 250°C, which was maintained for 5 min. The GC oven temperature program began at 35°C for 5 min, then increased to 50°C at a rate of 3°C/min, followed by a further increase to 250°C at a rate of 20°C/min, and was held at 250°C for 5 min. The transfer line temperature was also maintained at 250°C. The mass spectrometer was operated in an electron impact (EI) mode with an electron energy of approximately 70 eV, and a solvent delay of 1 min was set. Quantitative analysis of analytes was firstly performed on selective ion monitoring mode (SIM) using quantifying m/z of 56, 70, 80, 142, 68, 96, 110, 110, 96, 126, 152, 138 for Hexanal, Heptanal, Pentanal, Nonanal, Furan, Furfural, Furan 2‐ethyl‐5 methyl, Furan 2‐Propyl, 3‐Furaldehyde, 5‐Hydroxymethylfurfural, 1‐pentanone 2‐furanyl and 2‐Pentylfuran, respectively (Table S1) and was further confirmed in full scan mode. The compounds were identified by comparing their mass spectra with data from the NBS library of standard compounds. Standards were used to confirm the retention times and mass spectra of each compound, and a positive identification was made based on these results.

### 2.7 Matrix Matched Standard Calibration Curves and Linearity

The preparation of calibration standard solutions was done by serial dilution of 500 ng/g stock solution of each analyte standard and then spiking in the 5 g of sample matrix, followed by heating at 70°C for 45 min in order to eliminate any remaining compounds in the matrix. No signal was obtained at their respective retention time and then matrix matched standard calibration curves were generated for each compound, separately. Meanwhile, different standards concentrations were prepared in accordance with their prevalence in food systems according to previous literature (Batool et al. 2020; Wang et al. 2017). Calibration of different standards and their linear ranges are presented in Table 1. Moreover, an external calibration method was opted in order to quantify all analytes in the sample matrices in triplicate. The peak areas integration was done by using Mass Hunter Workstation Software (Version B.07.00, Agilent Technologies Inc. 2015). Moreover, their determination coefficients (*R* ^2^) (Table 2) were calculated by using linear regression model in Microsoft Excel (Version 2015, Microsoft).

**TABLE 1:** The representation of matrix (*Vitis vinifera*) matched calibration for each compound and their limit of detection (LOD) and limit of quantification (LOQ) from SPME‐GC‐TQ/MS.

Compounds	Linear range (ng/g)	Calibration curve		LOD (ng/g)	LOQ (ng/g)
		Equation	*R* ^2^		
Hexanal	0.5–150	1.5845*x* + 0.1654	0.9997	0.014	0.027
Heptanal	0.5–150	1.1041*x* + 0.1659	0.9992	0.018	0.054
Pentanal	0.5–100	1.3567*x* + 0.1458	0.9992	0.023	0.068
Nonanal	0.5–150	0.1658*x* + 0.1367	0.9991	0.032	0.096
Furan	0.5–250	0.1098*x* − 0.1348	0.9998	0.012	0.036
Furfural	0.5–250	0.1673*x* + 0.1135	0.9991	0.125	0.375
Furan 2‐ethyl‐5 methyl	0.5–150	0.1355*x* + 0.1287	0.9996	0.109	0.327
Furan 2‐Propyl	0.5–100	0.1983*x* + 0.1087	0.9994	0.142	0.426
3‐Furaldehyde	0.5–150	0.1983*x* + 0.1398	0.9990	0.105	0.315
5‐Hydroxymethylfurfural	0.5–250	0.1902*x* + 0.1148	0.9994	1.056	3.168
1‐pentanone 2‐furanyl	0.5–100	0.0583*x* − 0.1198	0.9997	1.095	3.275
2‐Pentylfuran	0.5–350	0.1329*x* + 0.1653	0.9992	1.560	4.680

*Note:* LOD = 3*σ/*m*, LOQ = 10*σ/*m* where *m* refers to calibration curve slope and *σ* refers to standard deviation of *y*‐intercept.

**TABLE 2:** The calculated % recovery by spiking different standards concentration into the matrix (*Vitis vinifera*) and repeatability (%) in terms of intra‐day and inter‐day precision from SPME‐GC‐TQ/MS.

Compounds	Recovery (%)			Repeatability (%)	
	0.5 ng/g	2.5 ng/g	5 ng/g	Intra‐day	Inter‐day
Hexanal	90.32 ± 0.93	89.54 ± 0.87	92.54 ± 0.89	5.34 ± 1.23	4.35 ± 1.72
Heptanal	94.54 ± 0.56	91.54 ± 0.49	89.40 ± 0.39	6.45 ± 1.05	3.78 ± 1.32
Pentanal	88.54 ± 0.95	94.34 ± 0.89	93.55 ± 0.93	4.35 ± 1.12	5.43 ± 1.98
Nonanal	90.56 ± 1.34	88.98 ± 0.98	92.46 ± 1.45	2.64 ± 1.84	6.32 ± 2.54
Furan	96.54 ± 0.89	95.34 ± 0.89	91.32 ± 0.64	1.38 ± 0.89	3.89 ± 1.87
Furfural	95.32 ± 0.49	94.63 ± 0.08	93.76 ± 0.59	2.89 ± 1.75	4.90 ± 1.78
Furan 2‐ethyl‐5 methyl	90.64 ± 1.23	89.64 ± 0.83	91.45 ± 1.09	1.98 ± 4.89	3.89 ± 0.89
Furan 2‐Propyl	92.54 ± 1.09	94.89 ± 1.12	95.43 ± 0.49	3.98 ± 0.78	2.78 ± 0.78
3‐Furaldehyde	95.32 ± 0.89	94.32 ± 0.89	92.32 ± 1.23	1.89 ± 1.78	7.34 ± 0.89
5‐Hydroxymethylfurfural	93.45 ± 1.38	90.53 ± 0.04	89.36 ± 0.89	3.89 ± 2.44	5.32 ± 1.76
1‐pentanone 2‐furanyl	87.54 ± 3.89	89.53 ± 1.02	88.45 ± 0.87	1.98 ± 0.78	2.67 ± 1.98
2‐Pentylfuran	89.56 ± 1.34	90.49 ± 1.98	89.78 ± 1.49	1.45 ± 0.49	2.59 ± 1.04

*Note:* %Recovery = add analyte (ng)/found analyte (ng) × 100; % Relative Standard Deviation (RSD) = SD/mean × 100; data represented standard deviation of *n* = 3, where *n* represents number of samples.

### 2.8 Method Validation

Different validation parameters were considered during analysis including linearity (*R* ^2^), recovery (%), precision in terms of relative standard deviation (% RSD), limit of detection (LOD) and limit of quantification (LOQ). Method repeatability was determined by triplicate measurements of pure standards at three concentration levels (0.5, 2.5 and 5 ng/g). The repeatability was expressed in terms of relative standard deviation (% RSD), divided into inter‐day and intra‐day precision (Table 2).

$$\%\text{Relative Standard Deviation}\;\left(\mathrm{RSD}\right)=\mathrm{SD}/\text{mean}\times 100$$

Meanwhile, the calculation of inter‐day precision was obtained by extracting and analyzing sample matrix for 6 days, consecutively. However, the intra‐day precision was obtained by analysis of sample matrix in triplicate in a single day.

The recovery analysis was performed by modifying our previously reported method (Batool et al. 2020). Samples were further heated at 70°C for 45 min to perform recovery analysis, reducing all volatiles in order to avoid any possible deviation. Afterwards, three concentrations of analytes were added in the sample supernatant followed by triplicate analysis. The recovery results of each analyte are elaborated in Table 2.

$$\%\text{Recovery}=\text{added analyte}\;\left(\mathrm{ng}\right)/\text{found analyte}\;\left(\mathrm{ng}\right)\times 100$$

Furthermore, calculation of LOD and LOQ was performed by spiking the standards into sample supernatant and three calibration curves were generated for each analyte, containing slope (*m*), followed by selection of appropriate *m* values and standard deviation of *y*‐intercept (σ) from their regression lines from one of the best calibration curve (Table 1).

$$\mathrm{LOD}=3^{*}\sigma /m,\mathrm{LOQ}=10^{*}\sigma /m,$$

where, *m* refers to calibration curve slope; *σ* refers to standard deviation of *y*‐intercept.

### 2.9 Polyphenolic Contents Determination

#### 2.9.1 Solvent Extraction

Methanol extraction was applied to extract polyphenolics from both fresh and dried grapes (Dróżdż, Šėžienė, and Wójcik 2018). To accomplish this, 25 mL of methanol was blended with 5 g of both fresh and dried grapes and homogenized for 3 min at room temperature. The mixture was then kept in the dark at 4°C for 15 h, and subsequently centrifuged for 15 min at 16,000 ***g***. The resulting supernatant was collected and transferred into amber vials. The procedure was repeated twice, and the pooled supernatants were stored at −20°C until analysis.

#### 2.9.2 Total Phenolic Contents

The Folin–Ciocalteu method is a colorimetric assay that is utilized to quantify the total phenolic content of both fresh and dried samples (Dróżdż, Šėžienė, and Wójcik 2018). The standard employed in this method was Gallic acid, and its absorbance was measured at 725 nm using a Carry 50 UV–Vis spectrophotometer (Varian Australia) in terms of gallic acid equivalents (GAE mg/100 g fresh weight).

#### 2.9.3 Antioxidant Activity Assay

The method utilized to evaluate the antioxidant activity of both fresh and dried samples was the DPPH assay, as described by Manzoor et al. (2019). The DPPH assay was chosen for this study due to its simplicity, high reproducibility, and wide acceptance in the scientific community as a reliable method for assessing free radical scavenging activity. It effectively captures the primary mode of antioxidant action, which is electron or hydrogen donation, reflecting the capacity of antioxidants to neutralize free radicals and combat oxidative stress. In this protocol, 2,2‐diphenyl‐1‐picrylhydrazyl (DPPH) was employed as a free radical, and sample's ability to scavenge this radical was determined. To carry out the assay, 100 μL of the extract was added to 2 mL of DPPH solution in methanol, and reaction mixture was incubated for 2 h at room temperature in the dark. The decrease in antioxidant activity was measured by reading the absorbance at 517 nm using a Varian Australia carry 50 UV–Vis spectrophotometer, and the percent inhibition of free radical scavenging activity was calculated using the following formula:

$$\%\text{Scavenging capacity}=\left[\left(\mathrm{Abs}\;\text{Control}\right)-\left(\mathrm{Abs}\;\text{sample}\right)/\mathrm{Abs}\;\text{Control}\right]\times 100.$$

### 2.10 Sugar Determination

Sugars were analyzed in fresh and dried grape samples through Ultra Performance Liquid Chromatography/Mass Spectrometry (UPLC/MS) using an Agilent 1290/maxis impact instrument. The system consisted of a binary solvent manager, sample manager, and a column heater equipped with a BEH amide column (50 mm × 2.1 mm, i.d., 1.7 μm particle size) and an Atomic Pressure Chemical Ionization (APCI) source. The mobile phase was comprised of 0.1% ammonia in water (A) and 0.1% ammonia in methanol (B) at a flow rate of 1 mL/min at a temperature of 30°C. The electrospray source settings were as follows: flow rate of 0.4 mL/min, drying heater temperature of 180°C, pressure of set nebulizer at 0.3 bar, temperature of APCI heater of 0°C, positive ion polarity, capillary voltage of 3500 V, and charging voltage of 2000 V. To extract sugars, 5 g of differently treated samples were extracted with 60 mL of ethanol (85%). The extract was then placed in a round flask, magnetically stirred on a water bath at 80°C for 25 min and repeated twice to dissolve all sugars inside it. The extract was filtered by Whatman filter paper No 2, and the residues were washed with 75% ethanol. Lead acetate (2 g) was added to the residue while heating in a water bath at 40°C for 10 min to precipitate proteins. The supernatants were then evaporated in a water bath at 70°C under reduced pressure after centrifuged at 4000 rpm for 25 min. Finally, residues were dissolved in acetonitrile mixture (98%) and Milli‐Q water in the volumetric flask, followed by microfiltration of extracts and standards performed by 0.22 μm syringe filter before injection into UPLC/MS.

### 2.11 Amino Acids Analysis

The amino acid profiling was carried out utilizing the Hitachi L‐8900 amino acid analyzer, following a standardized protocol. The initial step has involved preparing the samples by mixing an appropriate amount of dried sample with ultrapure water for 2 min. The amino acids were then extracted for 10–15 min at room temperature, and resulting solution was poured into a 25‐mL volumetric bottle. A 4‐mL sample solution of fixed volume was then placed in a centrifuge tube. Sulfosalicylic acid (15%) with a 4:1 ratio was added to the sample, followed by an even mixing. The mixture was refrigerated at 2°C–4°C for over 60 min and centrifuged at 10,000 rpm for 15 min. The supernatant was then centrifuged again at 10,000 rpm for 15 min. The samples were further diluted with 1:1 to 1:100 diluents, filtered through a 0.22–0.45‐μm filter, and put into a reagent bottle for analysis. Standards with similar concentrations of 2.00 nmol were prepared, and the calibration was performed at 280 nm and 520 nm. The retention time and standard calibration were used to measure the concentration of each amino acid present in the sample.

### 2.12 Fatty Acids Analysis

The determination of fatty acids (FAs) was carried out by GC‐TQ/MS followed by single injection per sample performed in split mode using split ratio of 50:1 on a DB Wax column (30 m × 0.25 mm × 0.25 μm, Agilent Technologies, America) to get separation. The Helium gas was chosen as carrier gas, with a flow rate of 1.0 mL/min. Meanwhile, both injection ports and detector temperature were adjusted at 250°C. The analysis involved conversion of fatty acids in the samples into Fatty acid methyl esters (FAMEs) following the AOCS Official Method (American Oil Chemistry Society 1997). To prepare the FAMEs, 100 mg of samples were dissolved in 6.00 mL of n‐hexane, followed by addition of 6.00 mL of 0.4 moL/L KOH/methanol reagent. The mixture was then heated in a water bath at 50°C for 15 min at 600 rpm. After centrifugation at 3500 rpm, the supernatant was collected and filtered through a 0.22 μm membrane filter before determination. The FAMEs composition was analyzed by peak area and identified by Mass fragments. Mass fragments were selected for the identification and confirmation, showing high abundance and specificity for each type of FAMEs. The saturated FAMEs possessed abundant fragments at 143.1 m/z. However, unsaturated long chain FAMEs possessed stronger fragmentation than unsaturated short chain FAMEs and saturated FAMEs. The quantification of saturated, mono‐unsaturated and poly‐unsatured FAMEs was performed by two transitions: quantification by precursor → identification by precursor → identifier transition. The quantification of saturated FAMAs was done with the transitions of 143.1 → 55.1 m/z. However, the 143.1 → 101.1 m/z containing mono‐unsaturated FA were quantified by 97.1 → 55.1 m/z, followed by 97.1 → 69.1 m/z containing polyunsaturated FAMEs by 79.1 → 51.1 m/z, respectively.

### 2.13 Statistical Analysis

All data analysis was conducted using Microsoft Excel and MassLynx 4.1 SCN 805 Software. Additionally, Origin 8.5 data analysis software was employed for generating graphics. IBM SPSS Statistics Version 26.0.1.1 Software was utilized to determine significant differences (*p* < 0.05) in triplicate data (mean ± SD, *n* = 3) via Duncan's Multiple Range Test.

## 3 Results and Discussion

### 3.1 Optimization of SPME and GC‐TQ/MS

In order to obtain the efficient analysis, we firstly optimized GC‐TQ/MS conditions for method development and then optimized SPME conditions for better extraction of analytes to be further confirmed on GC column.

#### 3.1.1 GC‐TQ/MS

The conditions of GC/MS were optimized by considering different GC parameters such as oven temperature, choice of capillary column, detector parameters, the desorption temperature, carrier gas flow and injection mode, in order to obtain optimal separation and maximum response of all analytes while analysis. The efficiency of two capillary columns, HP5 MS (30 m × 0.25 × 0.5 μm) and DB Wax (30 m × 0.25 × 0.25 μm) were compared to investigate their efficiency for separation and analysis. However, HP5 MS column has provided best separation results for our concerned analytes and was selected for the subsequent experiments. The HP‐5MS column provided the best results for volatile analysis due to its optimal polarity, thermal stability, and compatibility with mass spectrometry (MS). Its stationary phase, consisting of 5% phenyl and 95% dimethylpolysiloxane, is ideal for separating a wide range of volatile compounds, particularly non‐polar and slightly polar ones. The column's excellent thermal stability allows it to handle the high temperatures required for volatile separation, ensuring reliable performance and maintaining compound integrity. Additionally, HP‐5MS exhibits low column bleed, which enhanced the sensitivity and accuracy of MS detection by producing cleaner spectra with reduced background noise. Its versatility in separating diverse compound classes and delivering sharp, symmetrical peaks with high resolution further contributed to its effectiveness in analyzing complex mixtures of volatile compounds. These factors make HP‐5MS a highly suitable and reliable choice for volatile analysis in (GC‐TQ/MS). Meanwhile, split mode and spitless injection mode were also compared for transfer of analytes from inlet liner towards GC column, and obtained split GC mode (5:1) for providing better peaks with lesser lose of late eluting than splitless mode, that have shown drawbacks while optimizing trials and did not provided consistently accurate results. Afterwards, optimization of ramping flow of carrier gas was performed for condensing analyte bands in the column to improve sensitivity. Then desorption temperature was optimized till 250°C for desorbing compounds from the fiber, without observation of any remained carry over in the blank injection. The detector temperature and gas flow rate were also optimized in order to obtain the best response by direct injection of standards.

#### 3.1.2 SPME

A univariate approach was applied to optimize parameters of SPME. Each factor was carefully examined in order to obtain the optimal response of analytes under following applied conditions. The details of optimization of each factor are in the followings.

The efficiency of 75 μm CAR/PDMS and CAR/DVB/PDMS was reported by previous studies (Hu et al. 2016; Condurso, Cincotta, and Verzera 2018). However present study has found 50/30 μm CAR/DVB/PDMS fiber has given more efficient extraction of analytes with higher response. It might be due to efficient capturing ability of three combinations of carboxen, divinylbenzene and polydimethylsiloxane in the fiber with minimum diameter of 50/30 to capture even very small analytes such as furan (Batool et al. 2020). Hence, it was selected for subsequent experiments. Moreover, extraction temperature is also an important factor influencing SPME efficiency (Tan and Yu 2012). We have investigated effects of three different temperatures (40°C, 45°C and 50°C). However, our study has found the best extraction results for most of compounds with high peak area on 45°C and it was selected for further experiments. Meanwhile an appropriate extraction is necessary for achieving equilibrium of analytes distribution among three phases, matrix solution, head‐space vials as well as fiber coating (Tan and Yu 2012). Hence, different extraction times ranged from 20, 25, 30, 35 and 40 min were investigated for extraction, followed by achieving 25 min as best extraction time for all analytes. After 25 min, significant decrease was observed in the extraction efficiency, which might be due to competition between matrix solution and analytes. It is due to the fact that as time increase, head‐space vial area could be occupied by analytes making fiber adsorption bit competitive (Condurso, Cincotta, and Verzera 2018). Meanwhile, agitation is also important to enhance mass migration and to accelerate thermodynamic equilibrium. Therefore, stiring speed was also investigated from 450, 500, 550, 600, 650, 700, 750 and 850 rpm. However, 600 rpm was found an optimal speed for efficient extraction. It might be because relatively higher stiring speed could cause a decrease in the extraction quantity of analytes because of an unstable violent sample agitation (Hu et al. 2016). Moreover, salting out is an important phenomenon to enhance the analytes extraction. NaCl addition in the sample before starting the extraction process can improve the ionic strength of sample (Batool et al. 2020). Therefore, different concentrations of NaCl ranging from 10%, 15%, 20%, 25% and 30% (w/v) were added in the extraction process. However, 15% NaCl was selected as the optimized salt for achieving best extraction response of analytes. Hence, the best fiber (CAR/DVB/PDMS), salt concentration (15% NaCl), stiring speed (600 rpm), extraction temperature (45°C) and extraction time (25 min) were selected for the subsequent experiments.

### 3.2 Validation of Analysis

This optimized method has provided excellent linear relationship between peak area and concentration of 12 analytes with *R* ^2^ ranged from 0.9990 to 0.9998. The calibration was generated at linear ranges at 0.5–150 ng/g for hexanal, 0.5–150 ng/g for heptanal, 0.5–100 ng/g for pentanal, 0.5–150 ng/g for nonanal, 0.5–250 ng/g for furan, 0.5–250 ng/g for furfural, 0.5–150 ng/g for furan 2‐ ethyl‐ 5 methyl, 0.5–100 ng/g for furan 2‐Propyl, 0.5–150 ng/g for 3‐furaldehyde, 0.5–250 ng/g for 5‐hydroxymethylfurfural, 0.5–100 ng/g for 1‐pentanone 2‐furanyl and 0.5–350 ng/g for 2‐pentylfuran (Table 1). Meanwhile, obtained LOD and LOQ values are also illustrated in the Table 1. The LOD values for quantified compounds are ranged from 0.012 to 1.560 and LOQ values are ranged from 0.027 to 4.680. By reviewing previous literature studies (Wang et al. 2017; Condurso, Cincotta, and Verzera 2018; Hu et al. 2016), this study has achieved more sensitive analysis of these 12 analytes by obtaining satisfactory LOD and LOQ results. Moreover, method recoveries for each compound were obtained by spiking three concentrations of standards in the sample matrix and obtaining results for each analyte at three concentration levels (Table 2). The recoveries for 12 analytes were ranged from 87.54% ± 3.89% to 96.54% ± 0.89% at 0.5 ng/g, 88.98% ± 0.98% to 95.34% ± 0.89% at 2.5 ng/g and 88.45% ± 0.87% to 95.43 ± 0.49 at 5 ng/g. Our analysis has obtained most of recoveries results greater than 90% denoting efficient analysis of present method. Meanwhile, the obtained values of precision in terms of relative standard deviation (% RSD) are divided in Intra‐day and Inter‐day values ranged from 1.38 ± 0.89 to 6.45 ± 1.05 and 2.59 ± 1.04 to 7.34 ± 0.89, respectively (Table 2) proving the precision and accuracy of the present method.

### 3.3 Formation of Aldehydes and Furans Compounds During Convective Hot Air Drying

During convective hot air drying, aldehydes and furans were formed due to various chemical reactions at different temperatures (75°C, 70°C, and 65°C). Table 3A–C depicts the respective formation pathways and retention times of these compounds, while their respective spectra have been shown in Figures S3 and S5. Hexanal, heptanal, pentanal and nonanal have been shown as C6 compounds and aldehydes, also formed in minor concentration in the blended grapes, however, disappeared after some time and might have been converted into other compounds. Their major cause of formation is the oxidation of linoleic acid, which can produce aldehydes such as heptanal, (E)‐2‐heptenal, (E)‐2‐octenal, (E)‐2‐nonenal, and (E,E)‐2,4‐nonadienal (Meeting et al. 1994; Whitfield and Mottram 1992).

**TABLE 3:** Different compounds formed at 75°C, 70°C and 65°C (convective hot air drying).

(A) Compounds	Class	Reaction pathways	Retention time (min)	Fresh grapes		Analytes (ng/g) at temperature (75°C) and drying time (h)							
						1 h	3 h	5 h	7 h	9 h	11 h	13 h	15 h
Hexanal	Aldehyde	LO	5.72	nd	0.21	1.08	nd	nd	nd	nd	nd	nd	nd
Heptanal	Aldehyde	LO, UFAO	10.314	nd	0.98	2.01	1.02	nd	nd	nd	nd	nd	nd
Pentanal	Aldehyde	LO	3.01	nd	nd	nd	nd	0.89	nd	nd	nd	nd	nd
Nonanal	Aldehyde	UFAO	13.99	0.43	0.53	0.78	0.54	1.23	0.45	0.25	2.13	0.56	0.89
Furan	Furan	MR, CR, AA, LO	1.56	nd	nd	nd	nd	nd	nd	1.59	2.03	3.72	4.92
Furfural	Furan	MR, CR	7.86	nd	nd	nd	nd	nd	0.78	1.02	4.32	5.09	6.93
Furan 2‐ethyl‐5 methyl	Furan	MR, CR, AA	10.92	nd	nd	nd	nd	nd	nd	nd	nd	1.14	3.02
Furan 2‐Propyl	Furan	LO, MR	5.34	nd	nd	nd	nd	nd	nd	nd	nd	nd	0.96
3‐Furaldehyde	Furan	MR, CR	7.34	nd	nd	nd	nd	nd	nd	nd	0.52	1.34	2.09
5‐Hydroxymethylfurfural	Furan	MR, CR of 3‐DG	15.25	nd	nd	nd	nd	nd	nd	nd	nd	nd	4.98
1‐pentanone 2‐furanyl	Furan	MR	10.87	nd	nd	nd	nd	nd	nd	nd	nd	0.34	1.02
2‐Pentylfuran	Furan	LO, LO and AA interaction	12.51	nd	nd	nd	nd	nd	nd	nd	nd	nd	5.98

(B) Compounds	Class	Reaction pathways	Retention time (min)	Fresh grapes		Drying time (h) and temperature (70°C)								
Hexanal	Aldehyde	LO	5.72	nd	0.21	1.08	nd	nd	nd	nd	nd	nd	nd	nd
Heptanal	Aldehyde	LO, UFAO	10.314	nd	0.98	2.01	1.02	nd	nd	nd	nd	nd	nd	nd
Nonanal	Aldehyde	UFAO	13.99	0.43	0.53	0.56	0.34	1.01	0.45	0.25	2.21	nd	nd	nd
Furan	Furan	MR, CR, AA, LO	1.56	nd	nd	nd	nd	nd	nd	nd	1.06	2.34	2.04	2.28
2‐Furancarboxaldehyde (Furfural)	Furan	MR, CR	7.86	nd	nd	nd	nd	nd	nd	nd	nd	1.23	2.89	4.28
Furan 2‐ethyl‐5 methyl	Furan	MR, CR, AA	10.92	nd	nd	nd	nd	nd	nd	nd	nd	0.14	2.12	2.32
Furan 2‐Propyl	Furan	LO, MR	5.34	nd	nd	nd	nd	nd	nd	nd	nd	nd	nd	0.69
5‐Hydroxymethylfurfural	Furan	MR, CR of 3‐DG	15.25	nd	nd	nd	nd	nd	nd	nd	nd	nd	1.04	1.36
2‐Pentylfuran	Furan	LO, LO and AA interaction	12.51	nd	nd	nd	nd	nd	nd	nd	nd	1.21	3.13	3.65

(C) Compounds	Class	Reaction pathways	Retention time (min)	Fresh grapes		Drying time (h) and temperature (65°C)									
Hexanal	Aldehyde	LO	5.72	nd	0.21	1.08	nd	nd	nd	nd	nd	nd	nd	nd	nd
Heptanal	Aldehyde	LO, UFAO	10.314	nd	0.48	1.01	1.02	nd	nd	nd	nd	nd	nd	nd	nd
Nonanal	Aldehyde	UFAO	13.99	0.43	0.53	0.56	1.34	1.02	0.35	1.52	3.21	2.09	1.04	0.87	0.98
Furan	Furan	MR, CR, AA, LO	1.56	nd	nd	nd	nd	nd	nd	nd	nd	nd	nd	1.98	0.96
2‐Furancarboxaldehyde (Furfural)	Furan	MR, CR	7.86	nd	nd	nd	nd	nd	nd	nd	nd	nd	nd	1.48	2.98
Furan 2‐ethyl‐5 methyl	Furan	MR, CR, AA	10.92	nd	nd	nd	nd	nd	nd	nd	nd	nd	nd	nd	1.77
2‐Pentylfuran	Furan	LO, LO and AA interaction	12.51	nd	nd	nd	nd	nd	nd	nd	nd	nd	nd	0.45	1.38

*Note:* All compounds measured in ng; nd = not detected at limit of detection.

Abbreviations: 3‐DG, 3‐deoxyglucoson; AA, Amino Acid; CR, Ceramelization; LO, Lipid oxidation; MR, Maillard Reaction; UFAO, unsaturated fatty acids oxidation.

Meanwhile, during abrasive drying at relatively higher temperature (75°C), it was confirmed that furans and their derivatives could be produced during drying at latter stages, while obtaining a moisture reduction of more than 70%. Figure S1 shows the percent moisture reduction at different time intervals. The literature suggests that furan formation primarily occurs due to four processes: Maillard reaction, caramelization, degradation of ascorbic acid, and lipid oxidation (Owczarek, Meulenaer, and Scholl 2010; Yaylayan 2006). Caramelization typically requires higher temperature, so furan is more likely to initially form through the Maillard reaction. However, its rapid increase in the late drying period may be evidence of caramelization. It is likely due to the fruit being subjected, to long‐term heating (> 10 h) and having a very low moisture content (15% dry base). In previous study (Henry, William, and Korth 2010), it was observed that the onset of Maillard reactions occurred after 5–6 h of drying under similar conditions.

Moreover, formation of 5‐HMF at a later stage indicates that it might be produced from dehydration of 3‐deoxyglucoson. Other furanic compounds, such as 2‐pentylfuran, 1‐pentanone‐2‐furanyl, 3‐furaldehyde, furan 2‐ethyl‐5methyl were also detected at later stages of drying, suggesting that furan and some of its respective precursors might have changed their conformation into new compounds appeared in the dried product at later stages (Condurso, Cincotta, and Verzera 2018). Convective drying at 70°C showed that as the temperature decreased, the drying time increased, affecting analytes formation during different drying hours. Three compounds, hexanal, heptanal, and nonanal appeared in the blended grapes at the start of drying, however, disappeared in the later stages, possibly because they have changed into other compounds. However, there was not too much difference recorded in the formation of furan and other compounds when the temperature was dropped down to 70°C. However, only one compound, 1‐pentanone‐2‐furanyl, was missing during this dehydration process, possibly because it was produced in relatively lower concentration and was not detectable by the system as well as could not captured by SPME. It can be concluded that the concentration of other formed compounds was relatively lower at 70°C compared to 75°C (Table 3A,B). The convective drying process carried out at a low temperature of 65°C was associated with a longer drying time, and this greatly impacted formation of furanic compounds. The data presented in Table 3C showed that some compounds were not even produced, and concentration of furanic compounds was much lower compared to higher temperatures. Meanwhile, as mainly formed compounds in the later stages of drying, furan and furfural formation was particularly illustrated in Figure S5.

### 3.4 Formation of Compounds During Pulsed Electric Field (PEF) Pre‐Treatment

In order to improve drying time and enhance sensory properties of *Vitis vinifera* varieties, three types of PEF pre‐treatments were employed followed by drying, at a lower temperature of 65°C, which was found to be the most effective. Only the one lower temperature 65°C was chosen after applying three voltages of energy by PEF. The moisture reduction ratio has been shown in Figure S2. Meanwhile, PEF pre drying treatment results are shown in the Table 4A–C. Although the PEF pre‐treatment reduced drying time by up to 8 h, it also had an impact on the formation of various compounds. Some aldehydes were formed even at lower drying times during all PEF treatments, while the formation of furanic compounds such as 2(3H)‐Furanone, dihydro‐4,4‐dimethyl‐5‐(2‐oxopropyl), furan, 2‐furancarboxaldehyde, 1‐pentanone 2‐furanyl, 3‐furaldehyde indicated the occurrence of Maillard reaction and caramelization (Wang et al. 2017; Owczarek, Meulenaer, and Scholl 2010; Yaylayan 2006). These compounds were even formed earlier than in convective hot air drying, suggesting that the PEF pre‐treatment affects their formation directly.

**TABLE 4:** Different compounds formed at (65°C) with PEF pre‐treatment at 5 kV/cm, 3 kV/cm and 1 kV/cm before convective drying).

(A) Compounds	Class	Reaction pathways	Retention time (min)	Fresh grapes		Analytes (ng/g) at (65°C, 5 kV/cm) and drying time (h)							
						1 h	3 h	5 h	7 h	9 h	11 h	13 h	15 h
Hexanal	Aldehyde	LO	5.72	nd	0.21	1.08	nd	nd	nd	nd	nd	nd	nd
2‐Heptanal	Aldehyde	LO, UFAO	11.85	nd	0	nd	1.02	nd	nd	nd	nd	nd	nd
Pentanal, 5‐(methylenecyclopropyl)	Aldehyde	LO	13.48	nd	nd	nd	nd	nd	nd	nd	0.45	nd	nd
Benzene, 1‐methyl‐3(1‐methylethyl)	Aldehyde	MR, LO, UFAO	13.03	nd	nd	nd	nd	nd	0.45	0.25	nd	nd	nd
2(3H)‐Furanone, dihydro‐4,4‐dimethyl‐5‐(2‐oxopropyl)	Furan	MR, CR, AA, LO	14.50	nd	nd	nd	nd	nd	nd	nd	1.54	0.97	1.34
Furan	Furan	MR, CR	14.50	nd	nd	nd	nd	nd	nd	nd	nd	1.43	2.98
2‐Furancarboxaldehyde (Furfural)	Furan	MR, CR, AA	7.86	nd	nd	nd	nd	nd	nd	1.32	0.78	0.53	3.64
1‐pentanone 2‐furanyl	Furan	MR	5.34	nd	nd	nd	nd	nd	nd	nd	nd	1.54	2.43
3‐Furaldehyde	Furan	MR, CR	7.34	nd	nd	nd	nd	nd	nd	2.07	nd	nd	1.32

(B) Compounds	Class	Reaction pathways	Retention time (min)	Fresh grapes		Drying time (h) (65°C, 3 kV/cm)							
Hexanal	Aldehyde	LO	5.72	nd	0.51	1.08	nd	nd	nd	nd	nd	nd	nd
2‐Heptanal	Aldehyde	LO, UFAO	11.85	nd	0	nd	1.02	nd	nd	nd	nd	nd	nd
Benzene, 1‐methyl‐3(1 methylethyl)	Aldehyde	MR, LO, UFAO	13.03	nd	nd	nd	nd	nd	0.51	0.15	nd	nd	nd
2(3H)‐Furanone, dihydro‐4,4‐dimethyl‐5‐(2‐oxopropyl)	Furan	MR, CR, AA, LO	14.50	nd	nd	nd	nd	nd	nd	nd	0.74	0.82	0.43
2‐Furancarboxaldehyde (Furfural)	Furan	MR, CR, AA	7.86	nd	nd	nd	nd	nd	nd	nd	1.23	0.67	2.41
1‐pentanone 2‐furanyl	Furan	MR	5.34	nd	nd	nd	nd	nd	nd	nd	nd	0.72	1.38

(C) Compounds	Class	Reaction pathways	Retention time (min)	Fresh grapes		Drying time (h) (65°C, 1 kV/cm)								
Hexanal	Aldehyde	LO	5.72	nd	0.21	1.08	nd	nd	nd	nd	nd	nd	nd	nd
2‐Heptanal	Aldehyde	LO, UFAO	11.85	nd	0	nd	1.02	nd	nd	nd	nd	nd	nd	nd
Benzene, 1‐methyl‐3(1‐methylethyl)	Aldehyde	MR, LO, UFAO	13.03	nd	nd	nd	nd	nd	nd	0.21	0.35	nd	nd	nd
3‐Furaldehyde	Furan	MR, CR	7.34	nd	nd	nd	nd	nd	nd	nd	nd	nd	1.32	1.54
Furan	Furan	MR, CR	14.50	nd	nd	nd	nd	nd	nd	nd	nd	0.43	1.98	0.89

*Note:* All compounds measured in ng; nd = not detected at a limit of detection.

Abbreviations: 3‐DG, 3‐deoxyglucoson; AA, Amino Acid; CR, Ceramelization; LO, Lipid oxidation; MR, Maillard Reaction; UFAO, unsaturated fatty acids oxidation.

However, concentration of compounds produced during PEF pre‐treatment was lower than that of convective hot air drying, but still suggested that PEF pre‐treatment can provide activation energy to molecules inside the fruit matrix, leading to reactions even at lower temperatures. It was also observed that the Maillard reaction and sugars degradation was increased by this pre‐drying treatment because formation and concentrations of furans were observed in Table 4A–C. This study provides new insights into the effects of PEF pre‐drying treatment on furanic compounds formation. The formed concentrations of two mainly formed compounds, furan and furfural with different drying times and treatments are shown in Figure S6.

### 3.5 Total Phenolic Contents (TPC) and Antioxidant Activity (AA)

The phenolic profile is important for distinguishing between different fruit varieties such as mulberries (Perez‐Gregorio et al. 2011) and for the characterization of beverages, such as wines (Masa, Vilanova, and Pomar 2007), juices, and processing methods (Buyukkurt et al. 2019). In this study, six different treatments were used to dry grapes and their effects on the total phenolic contents (TPC) and antioxidant activity were compared to fresh samples (47.87 ± 1.12, 58.66 ± 1.32). It was found that as the treatment became more abrasive (such as drying at 75°C), the TPC and AA decreased significantly (*p* < 0.05) compared to less abrasive treatments like drying at 65°C. Results have shown decreased (27.54 ± 1.02, 41.78 ± 1.12) TPC and AA at 75°C as compared with 65°C (39.87 ± 0.79, 50.78 ± 1.12) for TPC and AA respectively (Table 5). The same trend was observed in pre‐drying experiments, where an abrasive pre‐drying treatment at 5 kV led to significantly (*p* < 0.05) decreased TPC and AA (35.76 ± 0.98, 48.98 ± 0.89) compared to pre‐drying treatment at 1 kV (39.09 ± 0.49, 53.05 ± 1.17) (Table 5). Phenolic compounds are mostly found in the skin and seeds of grapes. For instance, phenolic acids and flavonols are bound in grape skins, and so do flavan‐3‐ol derivatives in seeds (Souquet, Cheynier, and Moutounet 2000). Hence, different treatments can affects their extraction from the matrix, ultimately effecting the nutritional quality of dried product.

**TABLE 5:** Total phenolic contents and antioxidant activity.

	Control (untreated)	75°C (15 h)	70°C (17 h)	65°C (19 h)	PEF (5 kV/cm) (8 h)	PEF (3 kV/cm) (8 h)	PEF (1 kV/cm) (9 h)
Total phenolic contents (mg/100 g GAE)	47.87 ± 1.12^a^	27.54 ± 1.02^f^	34.11 ± 0.98^e^	39.87 ± 0.79^b^	35.76 ± 0.98^d^	38.73 ± 0.79^c^	39.09 ± 0.49^b^
Antioxidant activity (DPPH, %)	58.66 ± 1.32^a^	41.78 ± 1.02^f^	47.89 ± 1.03^e^	50.78 ± 1.12^d^	48.98 ± 0.89^e^	51.54 ± 1.16c	53.05 ± 1.17^b^

*Note:* Data presented in the same row for each compounds are statistically significant at *p* < 0.05 by Duncan multiple range test.

### 3.6 Changes in Amino Acids During Different Drying Treatments

Amino acids are building blocks of proteins and are integral to evaluate nutritional quality of fruits. This study analyzed 17 different free amino acids in grapes, including asparagine, threonine, serine, glutamine, proline, glycine, alanine, cystein, valine, methionine, isoleucine, leucine, tyrosine, phenylalanine, lysine, histidine, and arginine The aim was to observe changes in their concentration after undergoing drying treatments. Their respective retention time and concentrations have been given in Table 6. Previous studies either involved synthetic media or a single *Vitis vinifera* juice (Alegre et al. 2017) showed inconsistent correlations between amino acids and thiols (Pinu et al. 2014, 2019) after simple processing. The results showed that there were significant differences (*p* < 0.05) in the concentration of amino acids between fresh and treated samples (Table 6), indicating that chemical reactions may have occurred during hot air drying or PEF pre‐drying, resulting in a reduction in their concentration as they changed into other compounds. The Maillard reaction occurrence when amino acids reacted with sugars at high temperatures, may be one reason for these changes (Shen, Liu, and Jiang 2015). It was also observed that higher temperatures led to significantly lower concentrations of free amino acids after drying, suggesting that less abrasive treatments may help preserve the natural amino acid profile in the grapes which can ultimately enhance their nutritional quality.

**TABLE 6:** Changes in amino acids during different drying treatments.

Amino acid	Retention time (min)	Control (untreated)	75°C (15 h)	70°C (17 h)	65°C (19 h)	PEF (5 kV/cm) (8 h)	PEF (3 kV/cm) (8 h)	PEF (1 kV/cm) (9 h)
Asparagine	4.76	2.073 ± 0.98^a^	0.087 ± 0.98^e^	0	1.96 ± 0.09^b^	1.06 ± 0.54^d^	1.76 ± 0.09^c^	1.98 ± 0.53^b^
Threonine	5.40	7.46 ± 1.07^a^	3.166 ± 0.78^g^	4.19 ± 0.98^e^	5.82 ± 1.04^c^	4.08 ± 1.32^f^	5.09 ± 1.21^d^	6.89 ± 1.32^b^
Serine	5.99	6.05 ± 0.87^a^	2.27 ± 0.57^g^	3.74 ± 1.03^f^	4.53 ± 1.21^d^	3.87 ± 0.43^e^	5.10 ± 0.87^b^	5.07 ± 0.62^c^
Glutamine	6.72	5.89 ± 1.02^a^	2.09 ± 0.87^g^	2.17 ± 0.76^f^	2.46 ± 0.76^e^	2.54 ± 0.43^d^	2.89 ± 0.53^b^	3.61 ± 0.82^c^
Proline	7.49	29.31 ± 2.13^a^	12.59 ± 1.32^g^	19.32 ± 1.43^e^	24.08 ± 2.02^c^	17.32 ± 2.43^f^	21.85 ± 2.66^d^	25.31 ± 1.75^b^
Glycine	9.74	4.23 ± 0.76^a^	0.62 ± 0.08^b^	1.19 ± 0.34^b^	1.21 ± 0.03^b^	1.57 ± 0.43^b^	1.97 ± 0.98^c^	1.21 ± 0.53^b^
Alanine	10.60	17.28 ± 1.45^a^	11.09 ± 1.87^e^	13.23 ± 2.53^d^	14.98 ± 1.23^c^	11.87 ± 1.01^e^	14.78 ± 1.54^c^	15.89 ± 2.41^b^
Cysteine	11.69	2.112 ± 0.09^a^	0	0.10 ± 0.07^c^	0.11 ± 0.87^c^	0.06 ± 0.32^b^	0.09 ± 0.06^b^	0.11 ± 0.09^c^
Valine	12.34	2.63 ± 0.76^a^	0.92 ± 0.07^d^	1.73 ± 0.21^c^	2.12 ± 0.43^b^	1.78 ± 0.37^c^	2.41 ± 1.02^a^	2.57 ± 0.64^a^
Methionine	13.52	3.24 ± 0.87^a^	0	0.13 ± 0.05^d^	0.19 ± 0.21^d^	1.27 ± 0.09^b^	1.87 ± 0.72^b^	0.21 ± 0.32^c^
Isoleucine	15.81	1.22 ± 0.45^b^	0.93 ± 0.05^c^	1.16 ± 0.04^b^	1.20 ± 0.32^b^	1.53 ± 0.35^a^	1.98 ± 0.53^a^	1.21 ± 0.12^b^
Leucine	16.96	4.18 ± 0.21^a^	2.08 ± 0.98^c^	2.98 ± 1.02^c^	3.23 ± 0.65^b^	2.76 ± 1.01^c^	3.07 ± 1.01^b^	4.09 ± 1.02^a^
Tyrosine	17.86	2.40 ± 0.34^b^	1.05 ± 0.78^c^	2.10 ± 1.42^b^	2.32 ± 0.32^a^	1.32 ± 0.09^c^	1.78 ± 0.43^c^	2.08 ± 0.98^b^
Phenylanine	18.80	3.41 ± 0.43^a^	2.53 ± 0.54^b^	2.69 ± 0.76^b^	2.87 ± 0.67^c^	2.07 ± 0.78^d^	2.78 ± 0.67^c^	3.20 ± 0.91^a^
Lysine	21.11	2.93 ± 0.09^a^	1.19 ± 1.02^b^	1.34 ± 0.87^b^	1.65 ± 0.31^b^	0.59 ± 0.43^c^	0.96 ± 0.08^c^	1.09 ± 0.08^c^
Histidine	23.28	7.03 ± 0.63^a^	3.07 ± 0.98^e^	4.98 ± 1.02^d^	5.09 ± 1.04^c^	2.80 ± 0.85^d^	4.87 ± 1.03^d^	6.01 ± 1.03^b^
Arginine	27.18	33.89 ± 1.21^a^	12.64 ± 2.31^e^	17.82 ± 2.87^d^	21.87 ± 2.43^c^	20.18 ± 2.82^c^	25.98 ± 2.86^b^	27.67 ± 3.51^b^

*Note:* Data presented in the same row for each compounds are statistically significant at *p* < 0.05 by Duncan multiple range test.

### 3.7 Changes in Sugars Profiling During Drying Treatment

Fruit contains sugars such as fructose, glucose, and sucrose, playing a crucial role in the quality of dried products. However sugars can undergo changes due to caramelization or the Maillard reaction during the drying process. To better understand these changes, concentration of sugars was measured in fresh and dried samples that underwent different treatments. The results revealed that higher temperatures (75°C) and longer drying times resulted in significantly (*p* < 0.05) decreased concentrations of glucose, fructose, and sucrose (i.e., 9.23 ± 1.02, 15.83 ± 1.65, and 6.55 ± 1.05) in comparison to fresh samples (i.e., 22.78 ± 1.21, 34.98 ± 2.45, 14.32 ± 0.98), respectively (Table 7). Additionally, samples treated with PEF at different energy levels also showed reduced sugar concentrations, indicating the occurrence of chemical reactions during higher energy pre‐treatments. It has shown that the PEF treated samples with 1, 3 and 5 kV/cm even at same treatment temperature i.e., 65°C had significantly (*p* < 0.05) reduced concentration (11.32 ± 1.32, 17.98 ± 2.08, 9.53 ± 0.87) as compared to untreated samples (22.78 ± 1.21, 34.98 ± 2.45, 14.32 ± 0.98) for glucose, fructose and sucrose, respectively (Table 7). The findings suggest that careful control of drying conditions can help preserve the natural sugar profile in dried fruits.

**TABLE 7:** Changes in sugar content during different drying treatments.

Sugars	Retention time	Control (untreated)	75°C (15 h)	70°C (17 h)	65°C (19 h)	PEF (5 kV/cm) (8 h)	PEF (3 kV/cm) (8 h)	PEF (1 kV/cm) (9 h)
Glucose	3.14	22.78 ± 1.21^a^	9.23 ± 1.02^e^	9.32 ± 1.35^e^	13.42 ± 1.23^c^	11.32 ± 1.32^d^	14.53 ± 1.32^b^	16.98 ± 1.43^b^
Fructose	4.32	34.98 ± 2.45^a^	15.83 ± 1.65^d^	16.23 ± 1.07^d^	21.32 ± 2.09^b^	17.98 ± 2.08^c^	18.98 ± 1.72^c^	21.43 ± 2.04^b^
Sucrose	5.32	14.32 ± 0.98^a^	6.22 ± 1.05^f^	8.32 ± 1.78^e^	11.03 ± 1.56^b^	9.53 ± 0.87^d^	9.92 ± 0.89^d^	10.42 ± 1.06^c^

*Note:* Date presented in the same rows are statistically significant (*p* < 0.05) by Duncan multiple range test.

### 3.8 Fatty Acids Changes During Drying Treatment

Fatty acids are essential components of fruits and serve as the immediate substrate for the generation of ‘green leaves volatiles’ (GLVs), which are responsible for the fresh and green aroma of fruits (Chen et al. 2012; Kalua and Boss 2009). Hydroperoxides (HPOs), which are produced by lipoxygenase (LOX) on fatty acids, are metabolized by hydroperoxide lyase (HPL) into small molecules such as C6 alcohols, C6 aldehydes, and C6 esters (Gomez, Martinez, and Laencina 1995; Matsui 2006), which are the primary source of aroma in grapes and wines (Buttery, Turnbaugh, and Ling 1988). Grapes are especially rich in fatty acids, such as linoleic acid, hexadecanoic acid, palmitic acid, and stearic acid. The concentration of these fatty acids (*p* < 0.05) is influenced by heating and drying treatments (Table 8). As the drying time and temperature increase, the concentration of these fatty acids decreases significantly (*p* < 0.05), and they may be converted into other compounds. It has been demonstrated that the degradation of fatty acids, especially linoleic acid, can lead to the formation of furanic compounds (Shen, Liu, and Jiang 2015; Shen, Liu, and Jia 2016).

**TABLE 8:** Changes in fatty acids contents during different drying treatments.

Fatty acids	Retention time	Control (untreated)	75°C (15 h)	70°C (17 h)	65°C (19 h)	PEF (5 kV/cm) (8 h)	PEF (3 kV/cm) (8 h)	PEF (1 kV/cm) (9 h)
Hexadecanoic acid (C16:0)	11.45 ± 0.19	7.78 ± 0.12^a^	3.56 ± 0.08^e^	4.87 ± 0.04^d^	5.23 ± 0.08^c^	4.89 ± 0.08^d^	5.86 ± 0.12^c^	6.08 ± 0.11^b^
Linoleic acid (C18:2)	15.85 ± 1.08	11.42 ± 0.79^b^	7.43 ± 0.16^f^	5.73 ± 0.18^g^	8.65 ± 0.19^e^	8.32 ± 0.21^e^	9.83 ± 0.35^d^	10.02 ± 1.02^c^
Stearic acid (C18:0)	16.28 ± 1.12	4.89 ± 0.45^b^	2.67 ± 0.13^d^	3.68 ± 0.16^c^	3.78 ± 0.54^c^	3.07 ± 0.87^c^	3.78 ± 0.98^c^	3.89 ± 0.19^c^

*Note:* Date presented in the same rows are statistically significant (*p* < 0.05) by Duncan multiple range test.

## 4 Conclusion

Different drying treatments can adversely effect the nutritional quality of dried product which can ultimately lead formation of unwanted compounds health hazardous compounds. Therefore, a wise selection of drying method is needed to get minimum lose of nutritional quality of product. This study has optimized SPME‐GC‐TQ/MS and validated to evaluate the changes in different compounds formation while drying. Moreover, it has also evaluated the effect of different drying treatments on the fruit's constituents, including sugars, amino acids, fatty acids, total phenolic compounds, and antioxidant activity by applying different analytical methods. The results showed that the drying treatments such as convective hot air drying or PEF had varying effects on the fruit matrix profile. Some drying methods were abrasive and potentially led to higher concentrations of health‐hazardous compounds and also deteriorated the fruits nutritional quality. Additionally, the contents of amino acids, sugars, fatty acids, total phenolics, and antioxidants were significantly influenced by applying different drying treatments, showing the significant effect of these applied treatments on the fruits quality. These findings highlight the importance of selecting appropriate drying techniques that preserve the quality of the fruit while avoiding the formation of harmful compounds. This will ensure food safety and maintain the nutritional and health benefits of the fruit for consumers.

## Author Contributions

**Zahra Batool:** software (equal), validation (equal), writing – original draft (equal), writing – review and editing (equal). **Dur e Sameen:** data curation (equal), formal analysis (equal). **Ding Wen:** formal analysis (equal). **Shanshan Hu:** formal analysis (equal). **Ume Roobab:** formal analysis (equal). **Bing Li:** investigation (equal). **Rana Muhammad Adil:** formal analysis (equal), validation (equal), visualization (equal). **Bairong Shen:** funding acquisition (equal), investigation (equal), resources (equal), supervision (equal).

## Conflicts of Interest

The authors declare no conflicts of interest.

## Supporting information


Data S1.

## Data Availability

Data associated with this research is confidential and not shared.
