# Correction to “Enhanced Analysis of Carcinogens and Nutritional Profile of *Vitis vinifera* by Employing Pulsed Electric Field”

**DOI:** 10.1002/fsn3.70405

**Published:** 2025-06-03

## Abstract

Batool, Z., Sameen, D.e., Wen, D., Hu, S., Roobab, U., Li, B., Aadil, R.M., and Shen, B. (2024), Enhanced Analysis of Carcinogens and Nutritional Profile of *Vitis vinifera* by Employing Pulsed Electric Field. Food Sci Nutr, 12: 10576‐10591. https://doi.org/10.1002/fsn3.4590

The name of the seventh author is corrected from “Rana Muhammad Adil” to “Rana Muhammad Aadil.” The online version of this article has been corrected accordingly.

We apologize for this error.

Batool, Z., Sameen, D.e., Wen, D., Hu, S., Roobab, U., Li, B., Aadil, R.M., and Shen, B. (2024), Enhanced Analysis of Carcinogens and Nutritional Profile of *Vitis vinifera* by Employing Pulsed Electric Field. Food Sci Nutr, 12: 10576‐10591. https://doi.org/10.1002/fsn3.4590

The name of the seventh author is corrected from “Rana Muhammad Adil” to “Rana Muhammad Aadil.” The online version of this article has been corrected accordingly.

We apologize for this error.

