# Supplementary material for: Enhanced Analysis of Carcinogens and Nutritional Profile of Vitis vinifera by Employing Pulsed Electric Field
Source: Food Sci Nutr. 2024 Nov 18;12(12):10576–91. doi: 10.1002/fsn3.4590 (PMC11666904; doi:10.1002/fsn3.4590)
Supplement: Supplementary file 1 — Data S1. [file FSN3-12-10576-s001.docx]

**Table 1:** Presentation of quantifying (m/z) and qualifying (m/z) ions of each compound

| **Matrix (*Vitis Vinifera*)**    **Compounds** | **Quantifying Ion (m/z)** | **Qualifier Ion 1**  **(m/z)** | **Qualifier Ion 2**  **(m/z)** | **Retention Time**  **(min)** |
| --- | --- | --- | --- | --- |
| Hexanal | 56 | 72 | 82 | 5.72 |
| Heptanal | 70 | 55 | 86 | 10.314 |
| Pentanal | 86 | 44 | 58 | 3.01 |
| Nonanal | 142 | 57 | 82 | 13.99 |
| Furan | 68 | 39 | 0 | 1.56 |
| Furfural | 96 | 95 | 39 | 7.86 |
| Furan 2- ethyl- 5 methyl | 110 | 96 | 43 | 10.92 |
| Furan 2- Propyl | 110 | 81 | 53 | 5.34 |
| 3-Furaldehyde | 96 | 39 | 77 | 7.34 |
| 5-Hydroxymethylfurfural | 126 | 96 | 69 | 15.25 |
| 1-pentanone 2- furanyl | 152 | 110 | 96 | 10.87 |
| 2-Pentylfuran | 138 | 81 | 82 | 12.51 |

**Figure 1.** Moisture reduction ratio formation of convective hot air drying at different temperatures

*Data represented standard deviation of n=3 samples for each analysis

**Figure 2.** Moisture reduction ratio formation after PEF pre-treatment at different voltages

*Data represented standard deviation of n=3 samples for each analysis

**Figure 3.** GC-TQ/MS Spectra of aldehydes


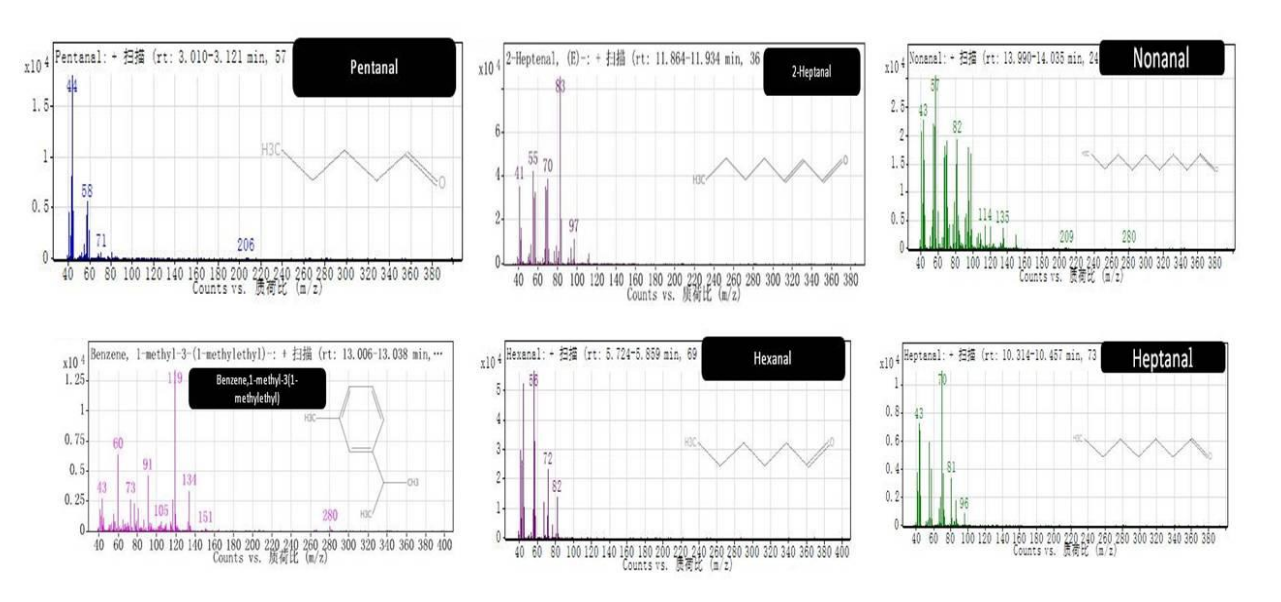


**Figure 4.** GC/TQ/MS Spectra of furan and its derivatives


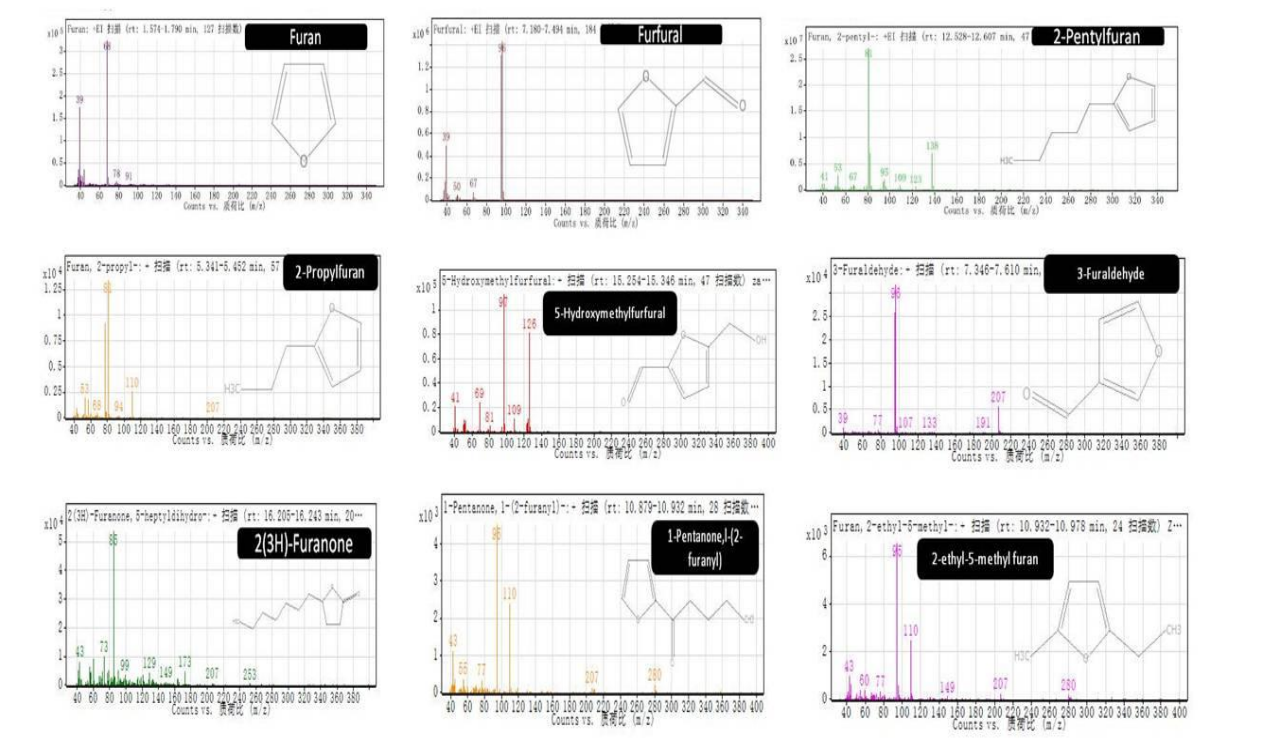


**Figure 5.** Evolution of furan and furfural as main functional compounds of drying time and temperature


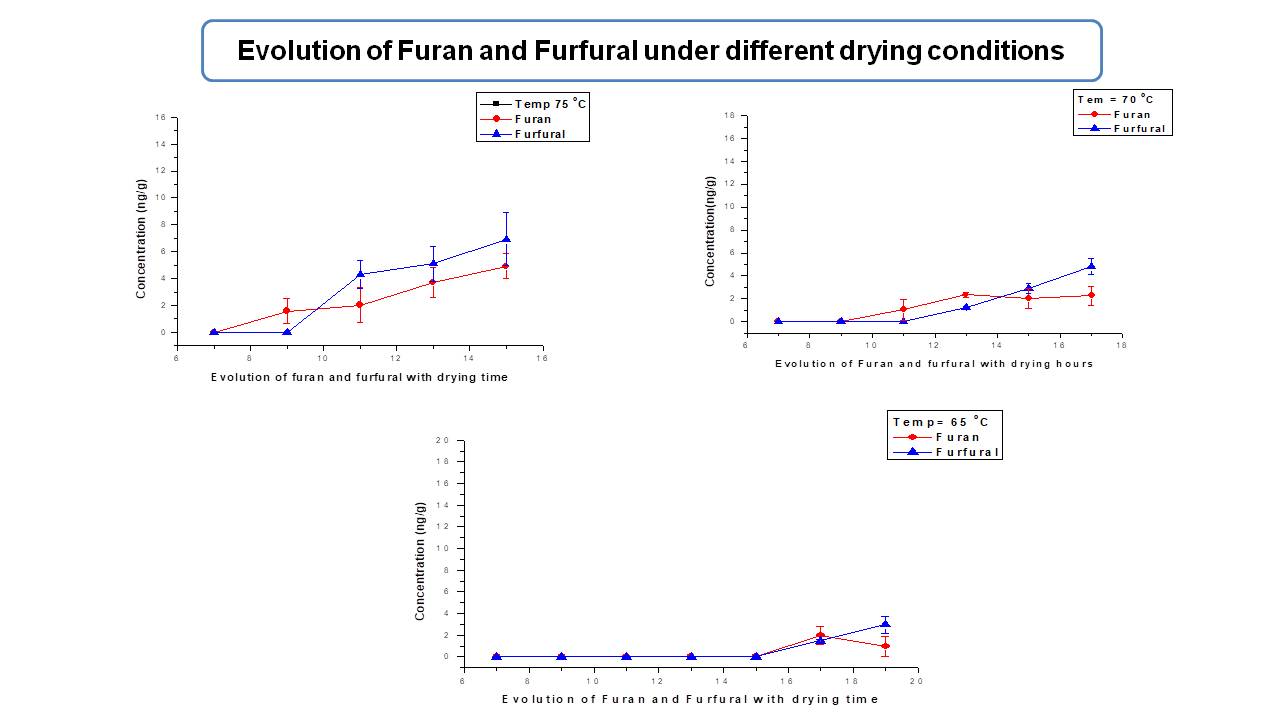


*Data represented standard deviation of n=3 samples for each analysis

**Figure 6.** Evolution of furan and furfural as main functional compounds of drying time and temperature with pre-drying effect


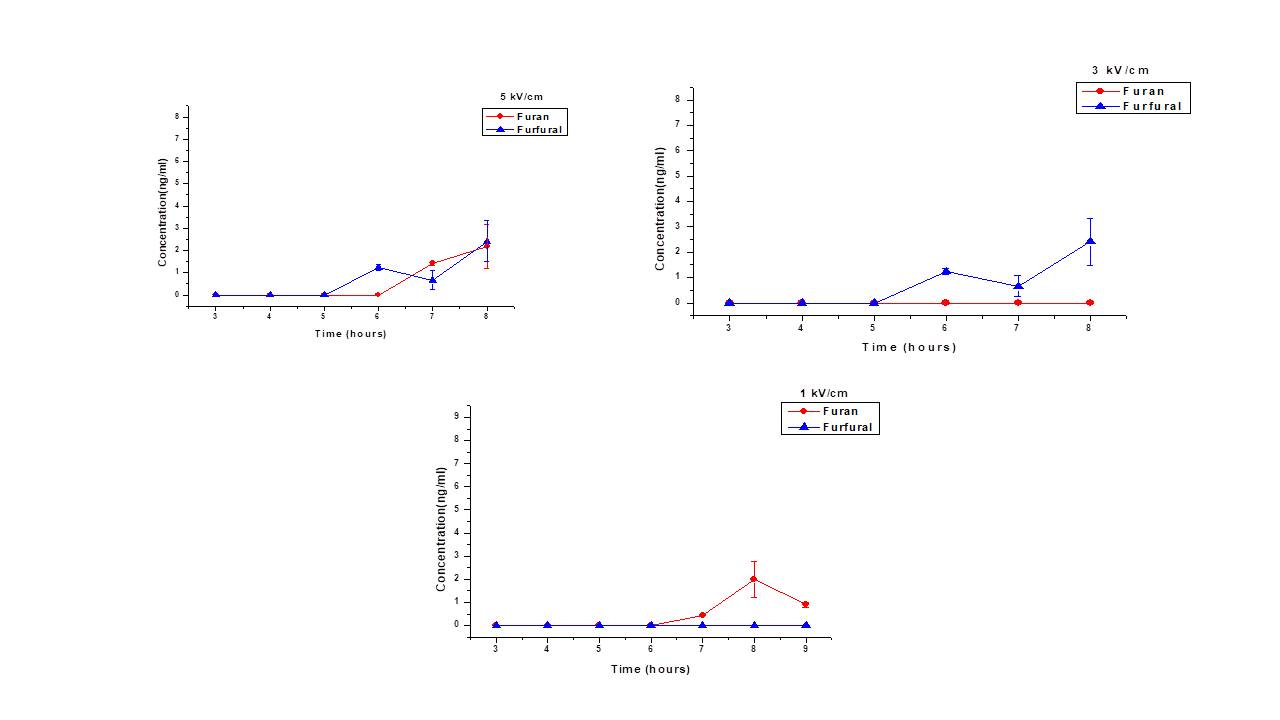


*Data represented standard deviation of n=3 samples for each analysis
